# Carnosic acid potentiates the anticancer effect of temozolomide by inducing apoptosis and autophagy in glioma

**DOI:** 10.1007/s11060-018-03043-5

**Published:** 2018-11-20

**Authors:** Naiyuan Shao, Jiahao Mao, Lian Xue, Rong Wang, Feng Zhi, Qing Lan

**Affiliations:** 1 Department of Neurosurgery, Third Affiliated Hospital of Soochow University, Changzhou, Jiangsu China; 2 Department of Neurosurgery, The Second Affiliated Hospital of Soochow University, #1055 Sanxiang Road, Suzhou, Jiangsu China; 3 Modern Medical Research Center, The Third Affiliated Hospital of Soochow University, #185 Juqian Road, Changzhou, Jiangsu China

**Keywords:** Carnosic acid, Temozolomide, Apoptosis, Autophagy, Glioma

## Abstract

**Objective:**

Malignant glioma is a lethal brain tumor with a low survival rate and poor prognosis. New strategies are urgently needed to augment the chemotherapeutic effects of temozolomide (TMZ), the standard drug in glioma treatment. Carnosic acid (CA) has been reported to have anticancer, antioxidant and anti-infectious properties. In this study, we aimed to investigate the anticancer effects and the underlying mechanisms of CA in combination with TMZ in glioma cancer cells.

**Methods:**

The glioma cancer cells were treated with TMZ, CA, or TMZ + CA. We evaluated cell survival by CCK-8 assay, cell anchorage-independent survival by colony formation assay, cell migration by wound-healing assay, cell cycle and cell apoptosis by flow cytometry, and protein expression by western blot.

**Results:**

CA enhanced the cytotoxic effect of TMZ in glioma cancer cells. CA enhanced TMZ-induced inhibition of colony formation and cell migration and enhanced TMZ-induced cell cycle arrest and cellular apoptosis. Immunofluorescence suggested that CA in combination with TMZ triggered autophagy. Furthermore, CA promoted TMZ-induced cell cycle arrest and cellular apoptosis by Cyclin B1 inhibition and activation of PARP and Caspase-3, while CA promoted TMZ-induced cellular autophagy by p-AKT inhibition, p62 downregulation and LC3-I to LC3-II transition.

**Conclusion:**

These data suggest that the combination therapy of CA and TMZ strengthens the anticancer effect of TMZ by enhancing apoptosis and autophagy.

## Introduction

Glioma, which is the most frequent primary tumor in the brain, accounts for almost half of all brain tumors in the United States and in China [1]. According to the World Health Organization (WHO) classification system, glioblastoma (GBM), the Grade IV glioma, is the most malignant glioma [2]. The current strategy for GBM is surgical resection followed by radiotherapy and adjuvant temozolomide (TMZ) chemotherapy [3]. Though significant improvement has been achieved in GBM therapeutic management, the patient 5-year survival rate is only 5.5% [1]. TMZ, an oral alkylating agent, is the first-line chemotherapy agent for glioma [4]. Its cytotoxicity results from inducing tumor cell apoptosis, autophagy and the unfolded protein response by alkylating DNA at the guanine residues [5]. One of the main causes for treatment failure is TMZ chemoresistance. Therefore, there is a great need to identify novel drugs with more curative effects and fewer side effects to promote sensitivity to TMZ in glioma treatment.

Carnosic acid (CA), a polyphenolic diterpene isolated from Rosemary (*Rosmarinus officinalis*) or common sage (*Salvia officinalis*), has various pharmacological effects, including antioxidant [6], anti-inflammatory [7], and anti-cancer properties [8]. For example, in hepatocellular carcinoma, CA significantly inhibited cell viability and enhanced apoptosis in vitro [9]. In cervical cancer, CA exerted anti-tumor activity by promoting apoptosis in vitro and in vivo through reactive oxygen species (ROS) production and JNK signaling pathway activation [10]. As in glioma, a previous study showed that CA at 27.5 µM reduced cell survival and induced cell apoptosis via proteasome-mediated degradation of several substrate proteins [11]. In addition to its capacities to directly inhibit tumor progression, CA could synergistically augment the activity of some chemotherapeutic agents in several different types of cancer. CA enhanced trastuzumab inhibition of cell survival and cell migration and induced cell cycle arrest in ERBB2^+^ breast cancer [12]. CA inhibited cell proliferation and enhanced cell apoptosis by increasing intracellular ROS in hepatocellular carcinoma [9]. The CA and fisetin combination treatment led to enhanced inhibition of cell growth by inducing apoptosis in lung cancer [13]. CA enhanced carmustine, lomustine, and β-lapachone-induced cell growth inhibition and cell cycle arrest in melanoma [14, 15]. However, the combination effects of CA and TMZ on glioma and the underlying molecular mechanism are still ambiguous.

In this study, we showed that a combination of CA and TMZ synergistically decreased cell viability, cell migration, and colony formation and induced cell cycle arrest by inducing cell apoptosis and autophagy in glioma cancer cells. The cytotoxicity of CA and TMZ co-treatment can be attributed to the downregulation of the PI3K/AKT pathway and the induction of apoptosis and autophagy. Taken together, these data show that the combination of CA and TMZ may provide a new therapeutic strategy for the treatment of glioma.

## Materials and methods

### Cell culture and materials

The glioma cell line U251 was purchased from the Chinese Academy of Science’s Cell Bank (Shanghai, China). The glioma cell line LN229 was kindly provided by Prof. Jun Cui at the School of Life Sciences, Sun Yat-sen University, Guangdong, China. The cells were grown in adherent conditions in DMEM supplemented with 10% FBS, 100 U/mL penicillin, and 100 mg/L streptomycin in a 5% CO_2_ incubator at 37 °C. CA and TMZ were purchased from Sigma Aldrich (St. Louis, MO, USA).

### Cell survival assay

The cells were seeded into a 96-well plate and incubated overnight at 37 °C. The cells were then incubated with CA, TMZ, or CA + TMZ at the indicated concentrations for 24 h, 48 h, and 72 h. Subsequently, each well was filled with 10 µL CCK-8 solution (Beyotime, Shanghai, China), and the plate was incubated for 4 h at 37 °C. The absorbance value was measured at 490 nm on EL×800 (BioTek, Vermont, USA). The experiment was repeated three times.

### Colony formation assay

The cells were trypsinized and seeded in 60 mm petri dishes containing 10% FBS DMEM. The cells were then treated with CA, TMZ, or CA + TMZ at the indicated concentrations and incubated for seven consecutive days. The cells were washed twice with PBS, fixed with 4% paraformaldehyde and stained by 0.1% crystal violet. The colonies with over 50 cells were scored under an inverse microscope (IX71, Olympus, Tokyo, Japan). The experiment was repeated at least three times.

### Scratch wound-healing assay

The cells were plated and grown in a 6-well plate until they reached > 80% confluence. A scratching wound was formed using a 200 µL sterile pipette tip in the middle of the cell monolayer. Cells were then washed with PBS to remove the debris and were cultured with complete medium containing CA, TMZ, or both at the indicated concentrations. The representative images were taken at 0 h and 24 h under an inverse microscope (IX71, Olympus, Tokyo, Japan).

### Cell cycle and apoptosis assays

The cells were seeded into 6-well plates and grown until > 80% confluence. The cells were then treated with CA, TMZ, or CA + TMZ at the indicated concentrations for 24 h. For cell cycle analysis, cells were trypsinized and fixed in 70% ethanol overnight. The cells were stained according to the manufacturer’s protocol using the cell cycle analysis kit (Beyotime, Shanghai, China) and detected by flow cytometry (Guava EasyCyte 6HT-2L, Merck Millipore, Darmstadt, Germany). For cell apoptosis analysis, the cells were stained with the cell apoptosis analysis kit (Beyotime, Shanghai, China) according to the manufacturer’s protocol and detected by flow cytometry (Guava EasyCyte 6HT-2L, Merck Millipore, Darmstadt, Germany).

### Immunofluorescence analysis

The cells were seeded on cover slips in a 6-well plate and then were treated with CA, TMZ, or both at the indicated concentrations for 24 h. The cells were fixed with 4% paraformaldehyde for 40 min under room temperature and were then stained with LC3 antibody (1:100, #2775, Cell Signalling Technology, MA, USA). The nuclei were counterstained with 0.1 µg/ml DAPI. Cells were then stained with a secondary antibody (1:1000, #A-11034, Thermo Fisher Scientific, MA, USA). The coverslips were viewed using confocal microscopy (FV10i, Olympus, Tokyo, Japan) with appropriate filters.

### Western blot analysis

The cells were cultured in a 6-well plate and were treated with CA, TMZ, or both at the indicated concentrations for 24 h. The cells were lysed in RIPA buffer (Beyotime, Shanghai, China) containing 1% protease and phosphatase inhibitor cocktail (Roche, Basel, Switzerland). Supernatant was collected after centrifuging at 12,000 rpm for 15 min at 4 °C. The protein concentration was determined using a Standard BCA Protein Assay Kit (Thermo Fisher Scientific, MA, USA). Western blot analysis was performed according to the standard protocol [16]. The primary antibodies against LC3 (#2775), Cyclin B1 (#4138), PARP (#9532), Caspase-3 (#9665), cleaved Caspase-3 (#9664), AKT (#4691), p-AKT (#4060), p62 (#8025), and β-actin (#3700) were purchased from Cell Signaling Technology (MA, USA). Chemiluminescent signals were detected using an ECL plus kit (Thermo Fisher Scientific, MA, USA) on a ChemiDoc Touch Imaging System (Bio-Rad, CA, USA).

### Statistical analysis

Data were expressed as the mean ± SD and were analyzed by GraphPad Prism 5.0 software. One-way analysis of variance (ANOVA) and two-tailed Student’s t-test were employed to compare different groups. p < 0.05 was considered statistically significant.

## Results

### CA and TMZ combination inhibited cell viability

**Fig. 1:**
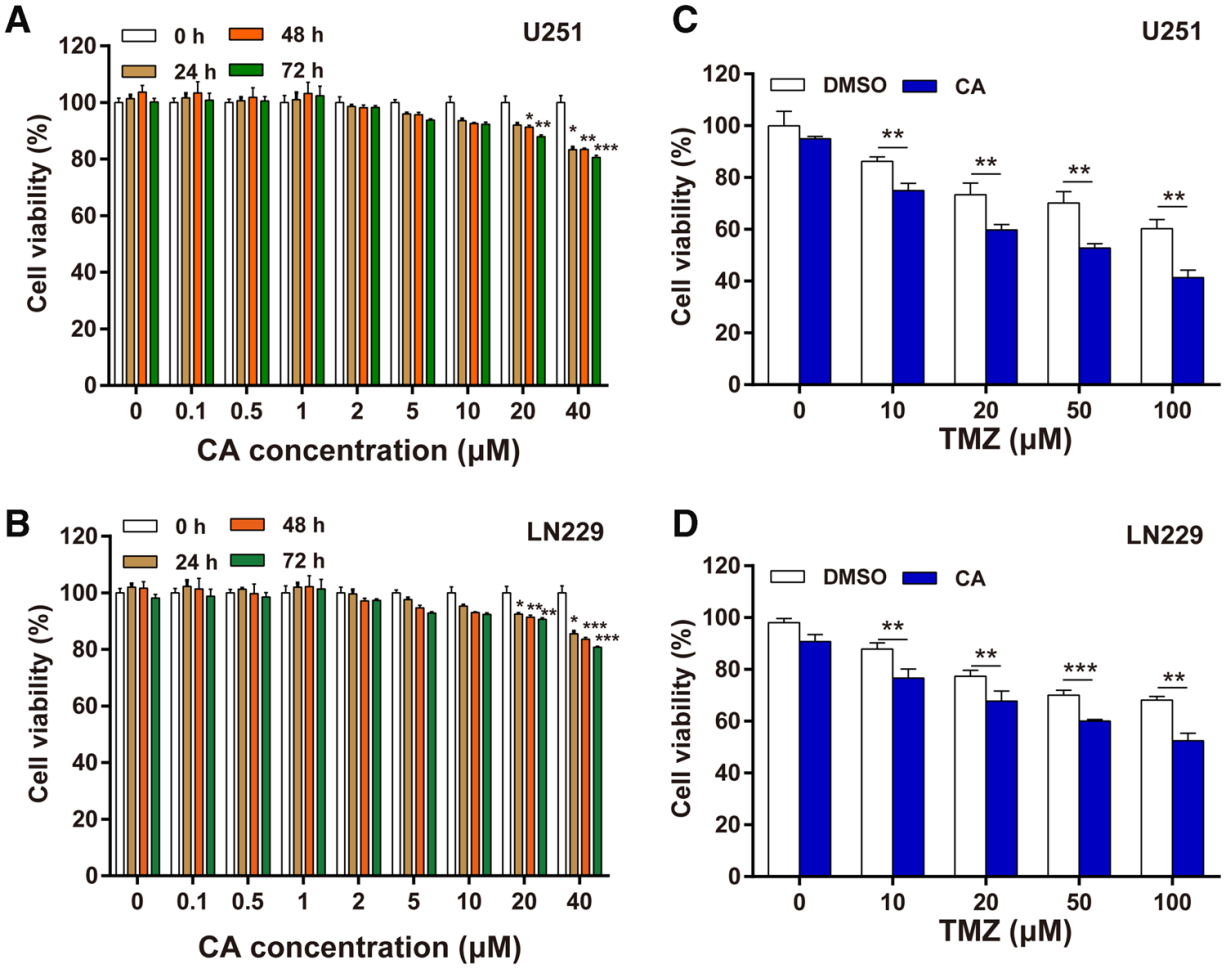
Effects of CA, TMZ, and CA + TMZ on cell proliferation. **a** The U251 cell viability was detected by CCK-8 assay after the cells were treated with the indicated concentrations of CA. **b** The LN229 cell viability was detected by CCK-8 assay after the cells were treated with the indicated concentrations of CA. **c** The U251 cell viability was detected by CCK-8 assay after different treatments with TMZ or CA + TMZ. **d** The LN229 cell viability was detected by CCK-8 assay after different treatments with TMZ or CA + TMZ. The results shown are representative of three different experiments. *p < 0.05, **p < 0.01, ***p < 0.001

To determine the antitumor effects of CA and TMZ combination in glioma, the U251 and LN229 glioma cancer cells were treated with CA, TMZ or TMZ + CA at indicated concentrations. As shown in Fig. 1a, b, no obvious cytotoxicity was observed in U251 cells as well as in LN229 cells after CA administration at the concentration of 10 µM or lower. Thus a CA concentration of 10 µM was selected in the subsequent experiments. As expected, treatment with TMZ (0–100 µM) significantly reduced cell viability in a dose-dependent manner in both U251 and LN229 cells (Fig. 1c, d). Moreover, after the combination treatment of CA with TMZ, the inhibitory effect of TMZ on U251 and LN229 cells was significantly increased (Fig. 1c, d). These results indicated that CA combined with TMZ has an enhanced antitumor effect in glioma.

### CA and TMZ combination inhibited cell colony formation and cell migration

**Fig. 2:**
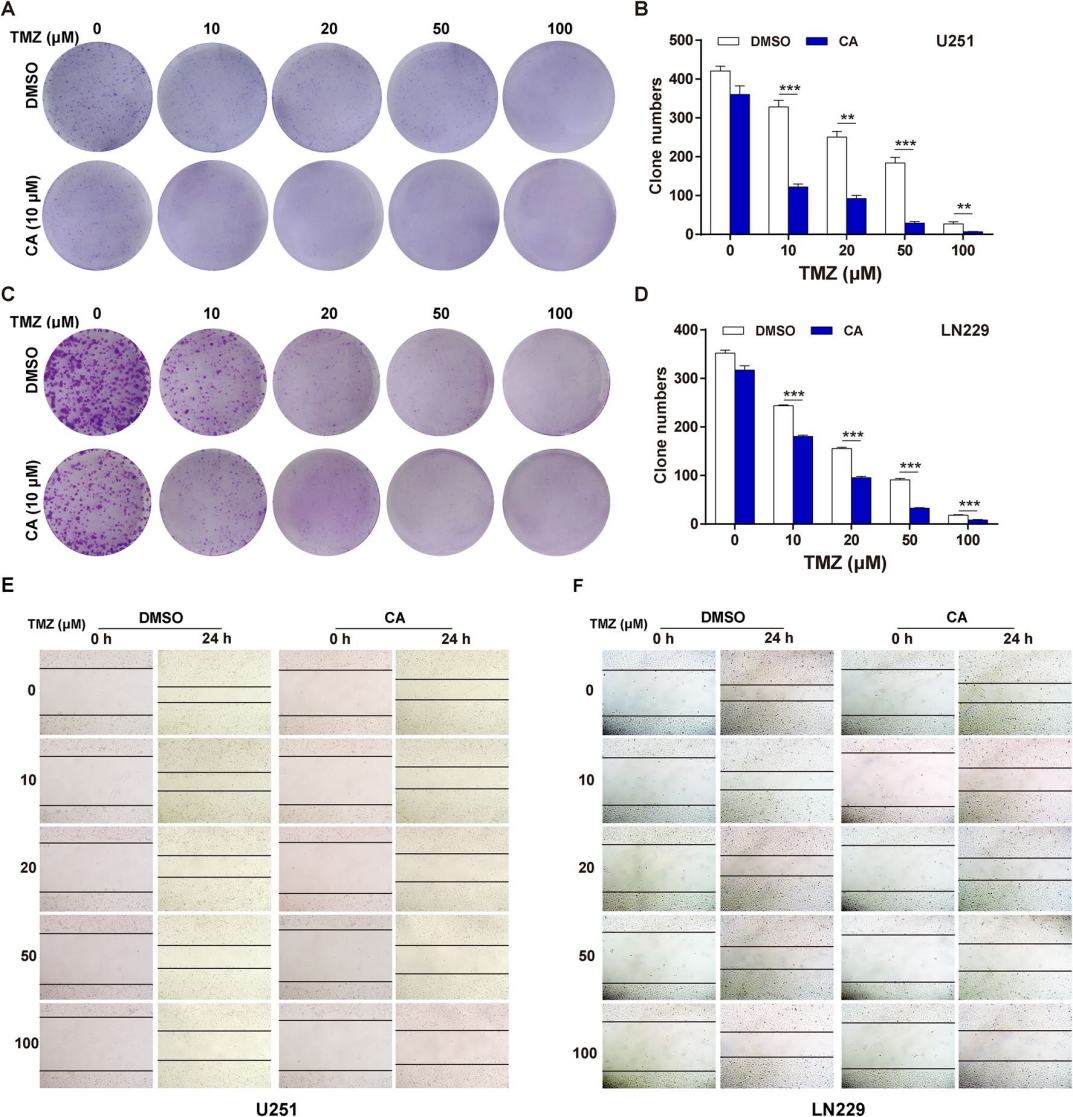
Effects of TMZ and CA + TMZ on cell colony formation and cell migration. **a** Representative images of U251 cell colonies after treatment with TMZ or CA + TMZ. **b** Statistical analysis of colony formation assay results on U251 cells. **p < 0.01, ***p < 0.001. **c** Representative images of LN229 cell colonies after treatment with TMZ or CA + TMZ. **d** Statistical analysis of colony formation assay results on LN229 cells. **p < 0.01, ***p < 0.001. **e** Representative images illustrating wound width after treatment with TMZ or CA + TMZ on U251 cells. The results shown are representative of three different experiments. **f** Representative images illustrating wound width after treatment with TMZ or CA + TMZ on LN229 cells. The results shown are representative of three different experiments

A colony formation assay was performed to evaluate cell division capabilities under different treatments. As shown in Fig. 2a, TMZ alone significantly inhibited cell clone formation in a dose-dependent manner, while co-treatment with CA prominently enhanced the inhibitory effect of TMZ on clone formation. The statistical analysis is shown in Fig. 2b. The inhibitory effects of CA and TMZ on cell clone formation were similar on LN229 cells (Fig. 2c) and the statistical analysis was performed in Fig. 2d. Next, a wound-healing assay was conducted to investigate the migration capability of cells following treatment. As shown in Fig. 2e, the speed of wound closure was significantly hindered by TMZ with increased concentration. The CA and TMZ combination showed enhanced effects in controlling the wound width of U251 cells. Similar results were observed in LN229 cells as shown in Fig. 2f. These results indicated that CA combined with TMZ has an inhibitory effect on glioma colony formation and cell migration.

### CA and TMZ combination arrested cell cycle progression and promoted cell apoptosis

**Fig. 3:**
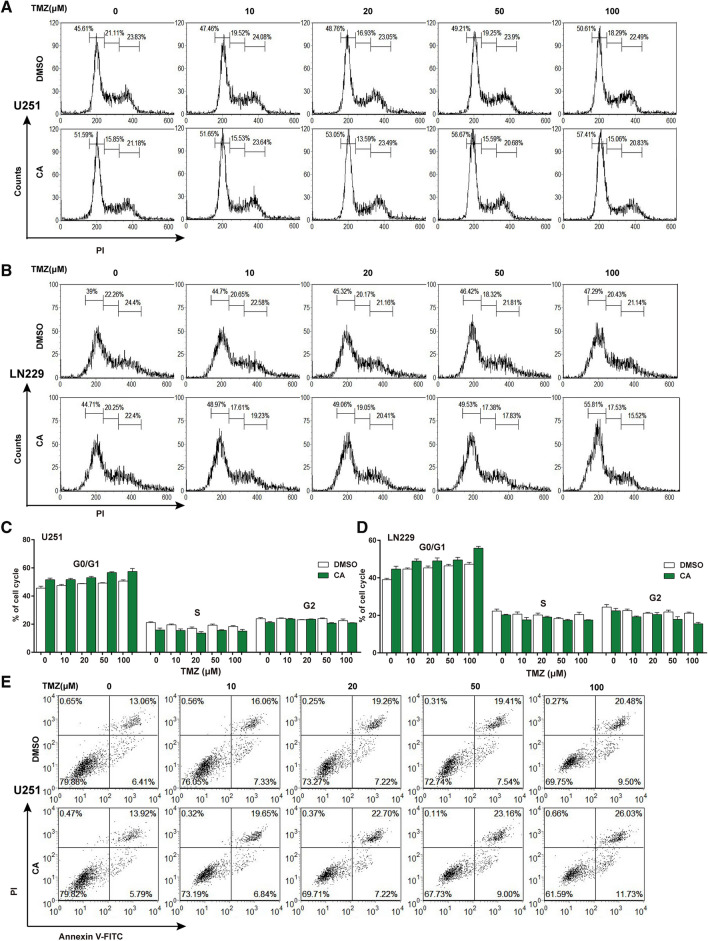

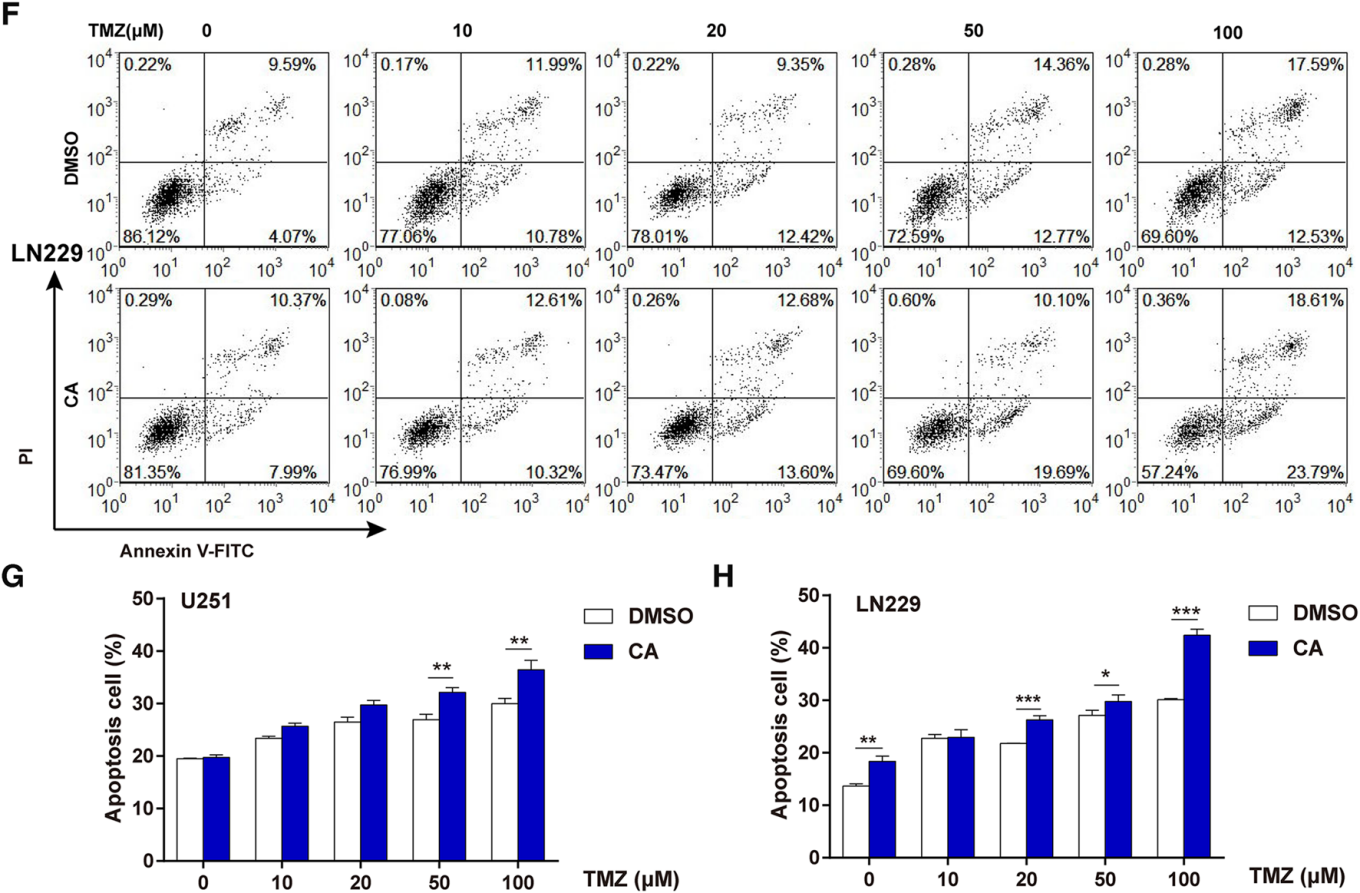
Effects of TMZ and CA + TMZ on glioma cell cycle and cell apoptosis. The cells were treated with TMZ or CA + TMZ at indicated concentrations for 24 h. The experiments were repeated for three times. **a** The cell cycle stage of U251 cells was evaluated by flow cytometry. **b** The cell cycle stage of LN229 cells was evaluated by flow cytometry. **c** Statistical analysis of the cell cycle assay results on U251 cells. **d** Statistical analysis of the cell cycle assay results on LN229 cells. **e** The cell apoptosis was evaluated by flow cytometry on U251 cells. The apoptosis rate = (Annexin V^+^PI^+^ + Annexin V^+^PI^−^)/total cells × 100%. **f** The cell apoptosis was evaluated by flow cytometry on LN229 cells. The apoptosis rate = (Annexin V^+^PI^+^ + Annexin V^+^PI^−^)/total cells × 100%. **g** Statistical analysis of cell apoptosis assay results from U251 cells. **p < 0.01. **h** Statistical analysis of cell apoptosis assay results from LN229 cells. *p < 0.05, **p < 0.01, ***p < 0.001

To explore the potential underlying mechanism by which CA could potentiate TMZ-induced cell death in glioma, cell cycle and cell apoptosis assays were performed and assessed by flow cytometry on U251 and LN229 cells. The cell cycle assay results showed that TMZ could induce a moderate G0/G1 phase accumulation and a slight S and G2/M phase reduction even at the highest concentration in our experiment, while the combination treatment could potentiate the effects induced by TMZ alone in both U251 and LN229 cells (Fig. 3a, b). The statistical analysis for the cell cycle assay on U251 cells is shown in Fig. 3c and that for LN229 cells is shown in Fig. 3d. The cell apoptosis assay showed that the rate of cellular apoptosis induced by TMZ increased in a concentration-dependent manner. The combination treatment had a higher apoptosis rate in both U251 and LN229 cells when compared with TMZ alone. In U251 cells, the apoptosis rate induced by 100 µM TMZ was 29.98% compared to an apoptosis rate of 37.76% when treated with TMZ and CA in combination (Fig. 3e). In LN229 cells, the apoptosis rate induced by 100 µM TMZ was 30.12% compared to an apoptosis rate of 42.40% when treated with TMZ and CA in combination (Fig. 3f). The statistical analysis for the U251 apoptosis assay is shown in Fig. 3g and that for the LN229 is shown in Fig. 3h.

**Fig. 4:**
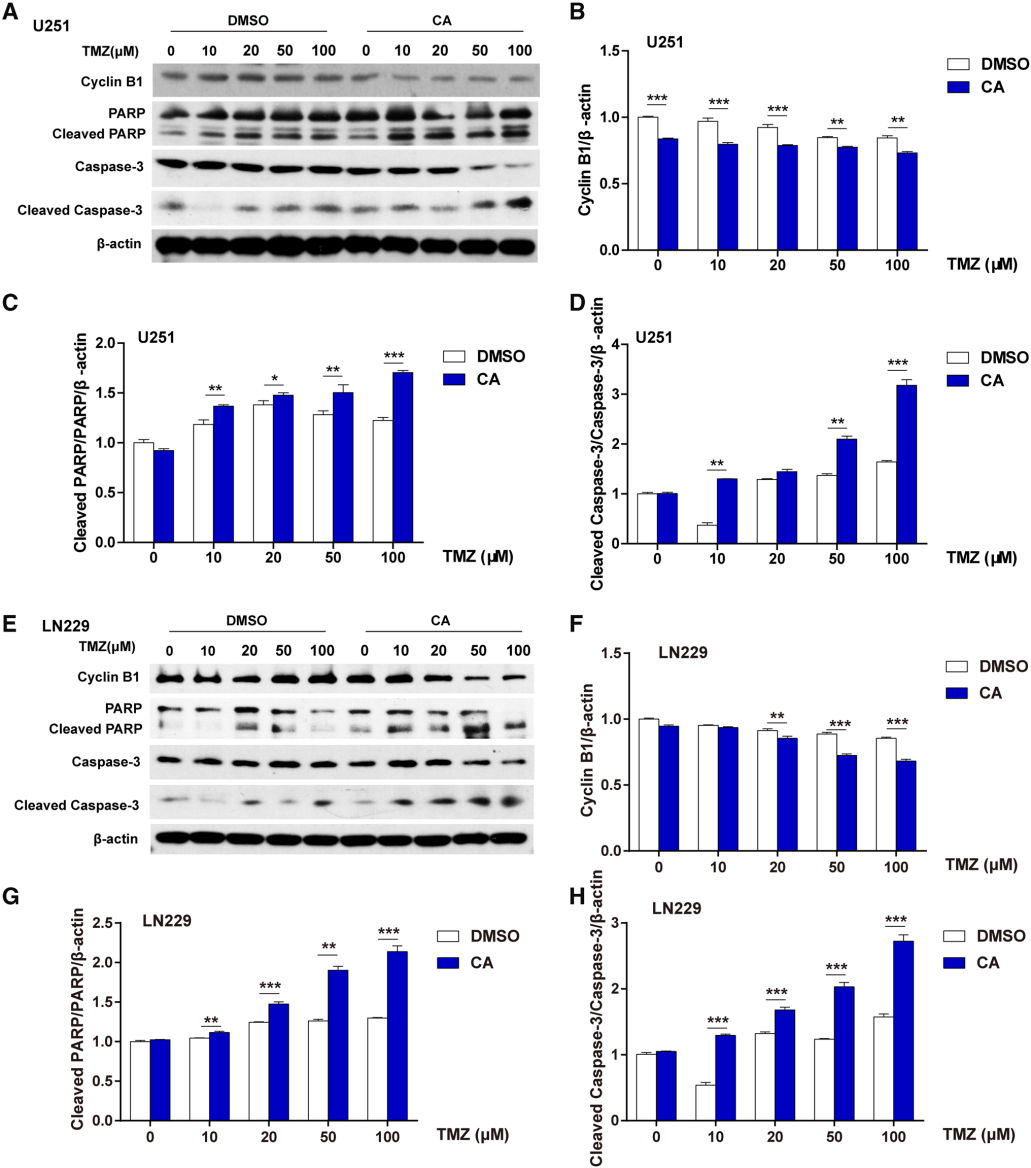
Effects of TMZ and CA + TMZ on glioma cell cycle and cell apoptosis related proteins. The cells were treated with TMZ or CA + TMZ at indicated concentrations for 24. **a** The protein levels of Cyclin B1, PARP, cleaved PARP, Caspase-3, and cleaved Caspase-3 in U251 cells were evaluated by western blot analysis. **b** Statistical analysis of Cyclin B1 protein level in U251 cells from western blot results. Relative Cyclin B1 protein level was determined by signal quantification and was normalized to β-actin. The relative Cyclin B1 protein level in the DMSO treatment group without TMZ or CA was arbitrarily set as 1. **c** Statistical analysis of cleaved PARP/PARP in U251 cells from western blot results. Relative cleaved PARP and PARP protein levels were determined by signal quantification and were normalized to β-actin. The ratio of cleaved PARP/PARP in the DMSO treatment group without TMZ or CA was arbitrarily set as 1. **d** Statistical analysis of the cleaved Caspase-3/Caspase-3 protein ratio in U251 cells from western blot results. Relative cleaved Caspase-3 and Caspase-3 protein levels were determined by signal quantification and were normalized to β-actin. The ratio of cleaved Caspase-3/Caspase-3 in the DMSO treatment group without TMZ or CA was arbitrarily set as 1. **e** The protein levels of Cyclin B1, PARP, cleaved PARP, Caspase-3, and cleaved Caspase-3 in LN229 cells were evaluated by western blot analysis. **f** Statistical analysis of Cyclin B1 protein level in LN229 cells from western blot results. Relative Cyclin B1 protein level was determined by signal quantification and was normalized to β-actin. The relative Cyclin B1 protein level in the DMSO treatment group without TMZ or CA was arbitrarily set as 1. **g** Statistical analysis of cleaved PARP/PARP in LN229 cells from western blot results. Relative cleaved PARP and PARP protein levels were determined by signal quantification and were normalized to β-actin. The ratio of cleaved PARP/PARP in the DMSO treatment group without TMZ or CA was arbitrarily set as 1. **h** Statistical analysis of the cleaved Caspase-3/Caspase-3 protein ratio in LN229 cells from western blot results. Relative cleaved Caspase-3 and Caspase-3 protein levels were determined by signal quantification and were normalized to β-actin. The ratio of cleaved Caspase-3/Caspase-3 in the DMSO treatment group without TMZ or CA was arbitrarily set as 1. The results shown are representative of three different experiments

To explore the potential molecular mechanism of CA-enhanced cell cycle arrest and cell apoptosis, western blot analysis was carried out. As shown in Fig. 4a, in U251 cells Cyclin B1 was slightly downregulated, while cleaved PARP and cleaved Caspase-3 were moderately increased when treated with TMZ alone. Furthermore, CA could potentiate these changes when CA and TMZ combination treatment was used. The statistical analysis for Cyclin B1, PARP, and Caspase-3 are shown in Fig. 4b–d, respectively. Similar results were observed in LN229 cells as shown in Fig. 4e–h. Taken together, these results indicated that CA and TMZ combination could arrest cell cycle and promote cell apoptosis in glioma.

### CA and TMZ combination induced autophagy

**Fig. 5:**
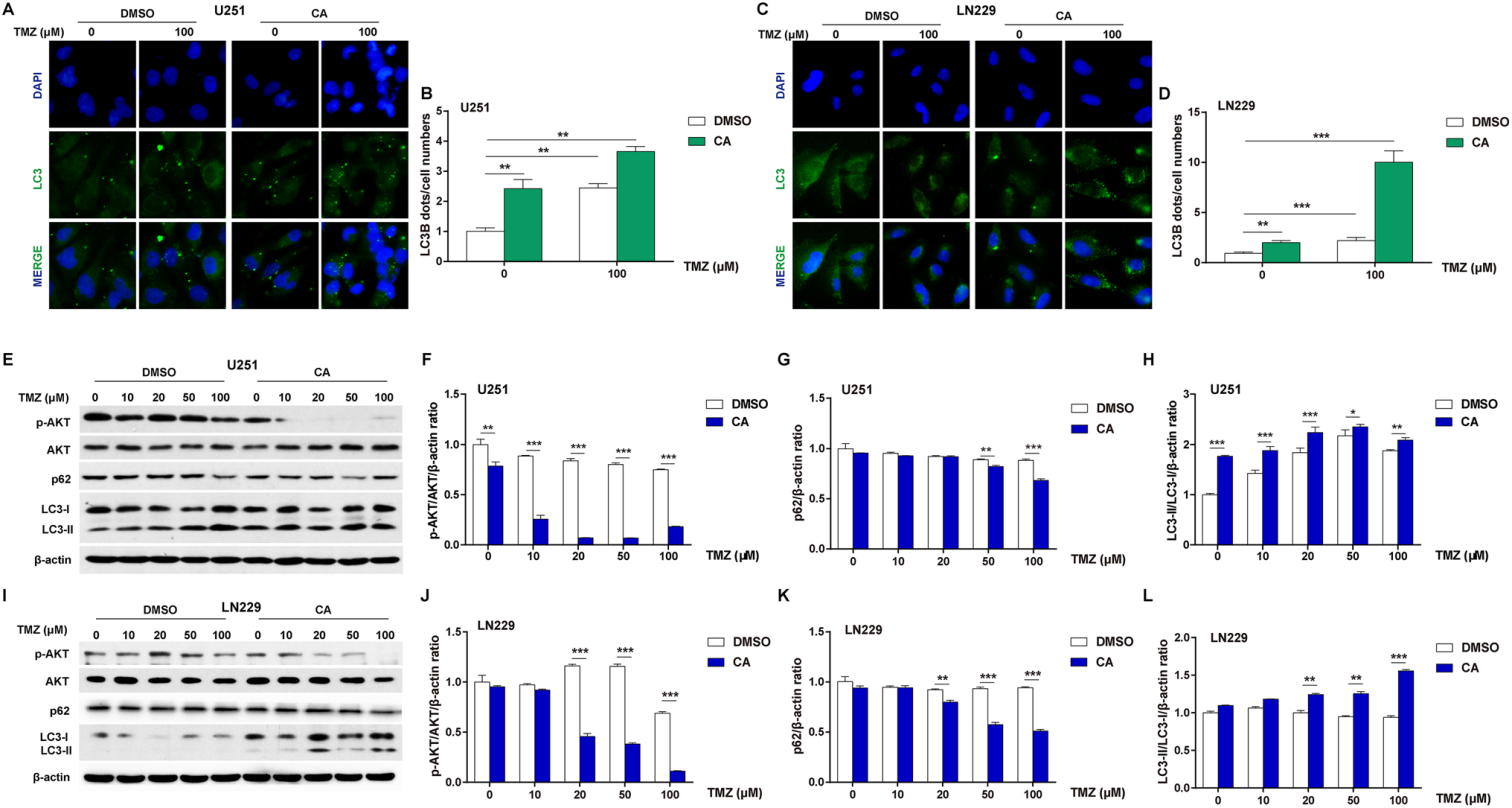
Effects of TMZ and CA + TMZ on glioma cell autophagy. The cells were treated with TMZ or CA + TMZ at indicated concentrations for 24 h. **a** The immunofluorescence analysis of LC3 in U251 cells. Nuclei were stained by DAPI. **b** The statistical analysis of number of LC3 dots per cell in U251 cells. **c** The immunofluorescence analysis of LC3 in LN229 cells. Nuclei were stained by DAPI. **d** The statistical analysis of number of LC3 dots per cell in LN229 cells. **e** The protein levels of p-AKT, AKT, p62, and LC3 in U251 cells were evaluated by western blot analysis. **f** Statistical analysis of p-AKT/AKT ratio in U251 cells from western blot results. Relative p-AKT and AKT protein levels were determined by signal quantification and were normalized to β-actin. The ratio of p-AKT/AKT in the DMSO treatment group without TMZ or CA was arbitrarily set as 1. **g** Statistical analysis of p62 in U251 cells from western blot results. Relative p62 protein level was determined by signal quantification and was normalized to β-actin. The relative p62 protein level in the DMSO treatment group without TMZ or CA was arbitrarily set as 1. **h** Statistical analysis of LC3-II/LC3-I in U251 cells from western blot results. Relative LC3-II and LC3-I protein levels were determined by signal quantification and were normalized to β-actin. The ratio of LC3-II/LC3-I in the DMSO treatment group without TMZ or CA was arbitrarily set as 1. **i** The protein levels of p-AKT, AKT, p62, and LC3 in LN229 cells were evaluated by western blot analysis. **j** Statistical analysis of p-AKT/AKT ratio in LN229 cells from western blot results. Relative p-AKT and AKT protein levels were determined by signal quantification and were normalized to β-actin. The ratio of p-AKT/AKT in the DMSO treatment group without TMZ or CA was arbitrarily set as 1. **k** Statistical analysis of p62 in LN229 cells from western blot results. Relative p62 protein level was determined by signal quantification and was normalized to β-actin. The relative p62 protein level in the DMSO treatment group without TMZ or CA was arbitrarily set as 1. **l** Statistical analysis of LC3-II/LC3-I in LN229 cells from western blot results. Relative LC3-II and LC3-I protein levels were determined by signal quantification and were normalized to β-actin. The ratio of LC3-II/LC3-I in the DMSO treatment group without TMZ or CA was arbitrarily set as 1

To determine the effect of CA and TMZ combination treatment on autophagy activation in glioma cancer cells, immunofluorescence was performed to detect LC3-II, an important component of the autophagosome. As shown in Fig. 5a, the combination treatment induced more coarse dots and punctate staining than with treatment of TMZ alone. The statistical analysis for this assay is shown in Fig. 5b. Similar results were observed in LN229 cells as shown in Fig. 5c, d. To reveal the molecular mechanism of this autophagy activation, some key autophagy—associated proteins were examined by western blot analysis. Obvious LC3-II accumulation and p62 reduction were observed in TMZ-treated U251 cells. Moreover, these phenomena were more apparent following CA addition (Fig. 5e). As the PI3K/AKT pathway is a key regulator in autophagy, the protein expression levels of p-AKT and AKT were detected. As shown in Fig. 5e, combination treatment significantly decreased the expression of p-AKT compared with TMZ alone in U251 cells. The statistical analyses for the levels of p-AKT/AKT, p62, and LC3-II/LC3-I are shown in Fig. 5f–h, respectively. Similar results were observed in LN229 cells as shown in Fig. 5i–l. There also existed some differences between U251 and LN229 cells when they were treated with CA, TMZ or both. For example, the ratio of the p-AKT/AKT in LN229 cells was not downregulated until the TMZ concentration greater than 20 µM. All the above results suggested that the combination of CA and TMZ could induce autophagy in glioma cancer cells.

## Discussion

The standard chemotherapy for glioma treatment with TMZ has some limitations, such as severe side effects and poor drug response [17, 18]. The need for novel drugs that can enhance TMZ bioavailability and overcome chemoresistance is urgent in clinical application. In this study, we demonstrated that the natural, polyphenolic drug CA may enhance the anticancer effect of TMZ by promoting the apoptosis and autophagy induced by TMZ.

CA has been widely proposed as a potential therapeutic compound in cancer treatment and prevention due to its ability to modulate cell growth and differentiation via different key molecules and signaling pathways [19]. CA impedes cell growth, induces cell cycle arrest, promotes apoptosis, and decreases mitochondrial membrane potential; CA has been shown to induce autophagy in various cancers: by enhancing p21 expression in melanoma [14] by inhibiting the AKT/mTOR pathway in hepatocellular carcinoma [20], by activating the JNK pathway in cervical cancer [10], by suppressing the Src/STAT3 pathway in renal carcinoma [21], by inhibiting the STAT3 pathway in colon cancer [22], by downregulating miR-15b in pancreatic cancer [23], and by modulating the AKT/IKK/NF-κB pathway in prostate carcinoma [24]. In glioma, Cortese et al. found that CA induced cell growth arrest and apoptosis via Cyclin B1, RB and SOX2 downregulation in glioblastoma [11]. CA may also function by enhancing the antitumor effects of current chemotherapeutic agents [19]. CA enhances the cellular cytotoxicity induced by β-lapachone in melanoma [15], by tamoxifen [25] and doxorubicin [26] in breast cancer, and by adriamycin [27] and arsenic trioxide [28] in leukemia. In this study, though TMZ could inhibit glioma cell growth, TMZ in combination with low-dose CA was more effective than TMZ alone. It is worth noting that CA at the concentration used in this work had little cytotoxic effect when used alone.

Apoptosis and autophagy are two distinct self-destructive processes that determine cell fate under physiological as well as pathological conditions [29]. Apoptosis is a process of programmed cell death that is executed by activated caspases and specific enzymes whose activation or inactivation lead to characteristic cell changes and cell death [30]. The apoptotic cascade is divided into the extrinsic apoptotic pathway and the intrinsic apoptotic pathway [31]. The extrinsic pathway is activated by death receptors and executed by initiator caspases and effector caspases (Caspase-3, Caspase-6, and Caspase-7) [32]. During drug-induced apoptosis, PARP is cleaved into fragments, and thus, cleaved PARP is usually used as a marker of apoptosis [33]. Our results showed that TMZ can increase the protein level of cleaved PARP and cleaved Caspase-3, while CA can enhance the effects of TMZ on PARP and Caspase-3 in glioma, indicating the potential of CA to induce apoptosis in glioma. However, it was worthy to note that the ratio of the cleaved Caspase-3/Caspase-3 in the 10 µM TMZ group was consistently lower than the other groups in our experiments. The underlying mechanism for this phenomenon still needs to be explored. Autophagy is the natural and orderly process to eradicate cytoplasmic organelles or unused proteins and thus to maintain cellular homeostasis [34]. As an important receptor in autophagy, LC3-II is considered the molecular marker for the autophagosome [35]. p62, a selective autophagy adaptor/receptor, binds with ubiquitinated proteins and LC3 for engulfment [36]. In our study, the combination treatment markedly increased the protein level of LC3-II and reduced the protein level of p62, suggesting that the autophagy induction was enhanced. Furthermore, the phosphorylation of AKT was decreased following CA treatment, suggesting that CA inhibits the PI3K/AKT signaling pathway, which is an important negative regulator of autophagy [37]. It is worth noting that there is a complex interaction between autophagy and apoptosis, especially in cancer etiology and treatment [38]. In our study, the CA and TMZ combination could induce apoptosis and autophagy in glioma cancer cells. However, the underlying mechanism of the connection between autophagy and apoptosis under the combination treatment remains unresolved. Our study may also have some limitations; for example, the signaling pathways influenced by CA were not exhaustively investigated. These issues can be resolved in our future studies.

In conclusion, the present study demonstrates for the first time that CA increases the sensitivity of glioma cancer cells to TMZ via the induction of apoptosis and autophagy. A combination therapy of TMZ with CA may be a promising therapeutic strategy for glioma in the future.
