# Correction to: Carnosic acid potentiates the anticancer effect of temozolomide by inducing apoptosis and autophagy in glioma

**DOI:** 10.1007/s11060-022-04006-7

**Published:** 2022-05-04

**Authors:** Naiyuan Shao, Jiahao Mao, Lian Xue, Rong Wang, Feng Zhi, Qing Lan

**Affiliations:** 1 Department of Neurosurgery, Third Affiliated Hospital of Soochow University, Changzhou, Jiangsu China; 2 Department of Neurosurgery, The Second Affiliated Hospital of Soochow University, #1055 Sanxiang Road, Suzhou, Jiangsu China; 3 Modern Medical Research Center, The Third Affiliated Hospital of Soochow University, #185 Juqian Road, Changzhou, Jiangsu China

## Correction to: Journal of Neuro-Oncology (2018) 141:277–288 10.1007/s11060-018-03043-5

There were errors in Figs. 2 and 5 in the original publication, as follows:

- In Fig. 2, the first author Naiyuan Shao submitted a preliminary version of Fig. 2 by mistake during the revision process. When he was arranging cell-wound images for Fig. 2F, he pasted some figures from already finished results in order to reserve proper position for those un-finished experiments, and he would replace these figures when the new results were got. In addition, y axis of Fig. 2B and Fig. 2D were not aligned and the texts such as TMZ, DMSO, and CA in Fig. 2E and Fig. 2F were not set at the same height. He had several preliminary versions of Fig. 2. But he falsely copied this preliminary version Fig. 2 to the revision version folder without check. The Fig. 2 was actually a preliminary version.
- In Fig. 5C, the figure for TMZ (100 μM) DAPI in the first row second column was the same as the figure for CA (0 μM) DAPI in the first row third column. This was an accident when the second author Jiahao Mao pasted the figures.
- In Fig. 5E, the first author Naiyuan Shao wrongly pasted the p62 western blot figure of LN229 to the position of the p62 western blot of U251, and then he pasted the Cyclin B1 western blot figure of LN229 to the p62 western blot figure of LN229. In the initial submitted version, the p62 western blot of U251 was still correct, but the p62 western blot of U251 was wrongly replaced in the revised version by the first author Naiyuan Shao.

The original article has been corrected.

